# Targeting the affective brain—a randomized controlled trial of real-time fMRI neurofeedback in patients with depression

**DOI:** 10.1038/s41386-018-0126-5

**Published:** 2018-06-23

**Authors:** David M. A. Mehler, Moses O. Sokunbi, Isabelle Habes, Kali Barawi, Leena Subramanian, Maxence Range, John Evans, Kerenza Hood, Michael Lührs, Paul Keedwell, Rainer Goebel, David E. J. Linden

**Affiliations:** 1 MRC Centre for Neuropsychiatric Genetics and Genomics, Division of Psychological Medicine and Clinical Neurosciences, School of Medicine, Cardiff University, Cardiff, CF24 4HQ UK; 2 Cardiff University Brain Research Imaging Centre, School of Psychology, Cardiff University, Cardiff, CF24 4HQ UK; 3 School of Allied Health Sciences, Faculty of Health and Life Sciences, De Montfort University, The Gateway, Leicester, LE1 9BH UK; 4 Pôle Hospitalo-Universitaire de Psychiatrie Adulte, Centre Hospitalier Guillaume Régnier, 108 Avenue du Général Leclerc, Rennes, 35703 France; 5 Centre for Trials Research, College of Biomedical and Life Sciences, Cardiff University, Cardiff, CF14 4YS UK; 6 Faculty of Psychology and Neuroscience, Maastricht University, Universiteitssingel 40, Maastricht, 6229 ER The Netherlands; 7 Brain Innovation B.V., Oxfordlaan 55, Maastricht, 6229 EV The Netherlands; 8 Centre for Affective Disorders, Institute of Psychiatry, Psychology & Neuroscience, King’s College London, 16 De Crespigny Park, London, SE5 8AF UK; 9 School of Mental Health and Neuroscience, Faculty of Health, Medicine and Life Sciences, Maastricht University, Universiteitssingel 40, Maastricht, 6229 ER The Netherlands

## Abstract

Functional magnetic resonance imaging neurofeedback (fMRI-NF) training of areas involved in emotion processing can reduce depressive symptoms by over 40% on the Hamilton Depression Rating Scale (HDRS). However, it remains unclear if this efficacy is specific to feedback from emotion-regulating regions. We tested in a single-blind, randomized, controlled trial if upregulation of emotion areas (NFE) yields superior efficacy compared to upregulation of a control region activated by visual scenes (NFS). Forty-three moderately to severely depressed medicated patients were randomly assigned to five sessions augmentation treatment of either NFE or NFS training. At primary outcome (week 12) no significant group mean HDRS difference was found (*B* = −0.415 [95% CI −4.847 to 4.016], *p* = 0.848) for the 32 completers (16 per group). However, across groups depressive symptoms decreased by 43%, and 38% of patients remitted. These improvements lasted until follow-up (week 18). Both groups upregulated target regions to a similar extent. Further, clinical improvement was correlated with an increase in self-efficacy scores. However, the interpretation of clinical improvements remains limited due to lack of a sham-control group. We thus surveyed effects reported for accepted augmentation therapies in depression. Data indicated that our findings exceed expected regression to the mean and placebo effects that have been reported for drug trials and other sham-controlled high-technology interventions. Taken together, we suggest that the experience of successful self-regulation during fMRI-NF training may be therapeutic. We conclude that if fMRI-NF is effective for depression, self-regulation training of higher visual areas may provide an effective alternative.

## Introduction

Depression has been recognized as the largest contributor to disability globally, and around one third of patients are thought not to respond to available treatments [1]. New efforts in the development of both pharmacological and non-pharmacological therapies are therefore needed. The last decade has seen considerable investment in the evaluation of innovative neuromodulatory therapies, particularly deep brain stimulation, but thus far this technique has not been superior to sham interventions [2].

Another way of targeting the brain directly is through neurofeedback training, a technique that has been increasingly explored to treat psychiatric conditions including depression [3, 4]. Neurofeedback can enable patients to develop personal strategies that are effective in self-regulating brain areas and networks associated with mental imagery through the feedback of signals that reflect their own neural activation patterns. Hence, the underlying principle of most neurofeedback protocols is supervised mental imagery training [5]. Mental imagery can be therapeutic for depression by increasing cognitive flexibility and capacity for positive mental simulation [6]. With neurofeedback patients’ engagement in mental imagery can be enhanced by monitoring and feeding back the associated brain activation.

In one pilot study, we demonstrated that patients suffering from mild to moderate depression learnt to upregulate brain areas using real-time fMRI neurofeedback (fMRI-NF). Target areas included the insula and lateral prefrontal areas, which were localized with positive affective visual stimulation. Moreover, only the group that had completed fMRI-NF training, but not a mental imagery only control group, experienced significant improvement in mood within four training sessions [7]. These findings have recently been corroborated in a double-blind, placebo-controlled randomized clinical fMRI-NF trial that used affective mental imagery training in unmedicated depressed patients. This trial found over 40% reduction in depressive symptoms in the intervention, but not in the placebo neurofeedback group [8]. However, the participants in the placebo group of that trial could not self-regulate their control target regions as well as the experimental group, suggesting that groups were not matched for the reward experience. Hence, it remains unclear whether the clinical efficacy of fMRI-NF in depression is specific to feedback from brain regions involved in affective processing, or whether the general experience of self-regulation may be in itself therapeutic.

We report the results from a randomized controlled trial (RCT) of fMRI-NF training in medicated patients with moderate to severe depression. The current trial compared upregulation of brain areas involved in emotion processing (NFE) with an active control procedure that involved upregulation of brain areas involved in higher visual processes (NFS). Specifically, both groups were provided with information that enabled patients to upregulate selected target areas. Whereas the NFE group was guided to imagine positive images similar to those seen in the respective blocks of the localizer run [7], the NFS group was guided to imagine relaxing scenes [9]. This design gave patients of the control group also the opportunity for self-regulation success and reward experience. We hypothesized that neurofeedback of emotion areas would produce clinical improvements exceeding those seen in the active control group.

## Method

### Patient recruitment

The study was approved in January 2012 by the South East Wales Research Ethics Committee and registered in February 2012 (NCT01544205). The first patient started the study in March 2012. All patients provided written informed consent. They were compensated for their time and travel costs in cash. Patients were recruited via general practitioner (GP) surgeries and Community Mental Health Teams (CMHTs) in South Wales and the National Centre for Mental Health (NCMH). To be included patients had to meet the following criteria: a diagnosis of unipolar depression, currently moderate or severe, confirmed with the Mini International Neuropsychiatric Interview (MINI), and current antidepressant treatment (with no change of dose in the preceding three months). We excluded psychotic symptoms, current substance dependence, eating disorders, claustrophobia and other MRI contraindications, and ongoing non-pharmacological treatment.

### Randomization and masking

Patients were randomly assigned to one of two groups using an adaptive randomization protocol developed by the South East Wales Trials Unit (SEWTU). The randomization protocol allocated patients to two groups, minimizing for differences in age, gender, duration of illness, medication type (with three categories: SSRI only; non-SSRI antidepressant; combination treatment) and baseline depression severity as measured with the Hamilton Depression Rating Scale (HDRS-17). After the patient had consented and completed all baseline measures, these were entered in a computer program (scripted in Microsoft Excel) and an allocation provided to the investigators conducting the study. Investigators running the MRI sessions needed to know group allocation in order to run the appropriate imaging protocols, but those conducting the assessments were blind to group allocation.

### Trial design

The intervention in both groups consisted of five training sessions, starting with four weekly sessions followed by a consolidation session after a break of 1 month to test if patients retained the ability to self-regulate target (regions of interest) ROIs. The third session served as transfer session during which no neurofeedback was provided to test if patients can upregulate target ROIs based on successful strategies learned during the previous two neurofeedback sessions. The last neurofeedback session occurred approximately 4 weeks after the 4th session to test if patients retained similar upregulation success. The actual duration of the intervention period was on average 12 weeks because of the need to schedule sessions around patients’ availability. Before the randomization, baseline measures of all clinical outcomes were recorded. Clinical measures were recorded again after the fifth neurofeedback session (week 12), and at follow up (FU; on average week 18 after start of the intervention). All patients who completed the trial received verbal debriefing at FU.

### Clinical and psychometric measures

The primary outcome measure was group mean difference in the Hamilton Depression Rating Scale (HDRS-17) [10] at the end of the intervention (session 5), which was administered by a clinician who was blinded to treatment group. Secondary clinical outcome measures were the group difference in the HDRS at follow-up and the group differences at both time points in the Hospital Anxiety and Depression Scale (HADS) [11] anxiety and depression subscales, the Quality Of Life scale (QOLS) [12] and EuroQol research foundation questionnaire (EQ-5D-5L), which assessed the subject’s health utility. The following self-rated psychometric measures were also acquired before and after the intervention and at follow-up: thought Control Ability Questionnaire (TCAQ) [13] to measure the perceived ability to control unwanted, intrusive thoughts, Thought Control Questionnaire (TCQ) [14] to measure the effectiveness of strategies used for the control of unpleasant and unwanted thoughts, the Self-Efficacy Scale (SES) to measure optimistic self-beliefs to cope with difficult life demands, with subscales for General Self-Efficacy (GSE) and Social Self-Efficacy (SSE) [15], the Behavioral Inhibition System and Behavioral Activation System (BIS/BAS) [16] to assess approach and avoidance motivation.

### Power calculation

The data from our pilot study [7] yielded an effect size (Cohen’s *d*) of 1.5 for the group difference in HDRS-17 improvement between active neurofeedback and a control intervention. Expecting a slightly more conservative effect size of 1.2, we estimated that a sample size of 15 patients in each group would achieve > 80% power for post-hoc *t*-tests (Bonferroni corrected, alpha-level 0.025, two-sided) and set a recruitment target of 40 patients to allow for 25% attrition. Although this effect size is unusual for add-on medication treatments, similar effect sizes have been reported for some non-pharmacological interventions [17], as well as for fMRI-NF compared to sham [8]. Furthermore, because fMRI-NF is still in the early phases of clinical evaluation and there is no agreement on the most suitable comparators for clinical trials it seemed prudent to take logistic and economic factors into consideration when setting up the trial.

### MR image acquisition and neurofeedback training

A 3 Tesla whole-body MRI system (General Electric, Milwaukee, USA) with an 8-channel head coil was used at the Cardiff University Brain Research Imaging Centre (CUBRIC). To identify target ROIs for neurofeedback training, each session began with a functional localizer (325 volumes, first 6 volumes discarded to ensure T1 equilibrium magnetization). An echo planar imaging (EPI) sequence (TR = 2 s, TE = 45 ms, flip angle = 80°, 30 slices, field of view = 192 mm, image matrix 64×64, in plane voxel size = 3×3 mm, slice thickness = 4 mm, gap of 1 mm) was used for fMRI, and a high-resolution 3D T1-weighted image (TR 7.9 s, TE 3.0 ms, TI 450 ms, flip angle 20°, matrix size 256×256, 1 mm isotropic voxel resolution) was acquired for anatomical co-registration. To control for physiological confounding factors of the BOLD signal [18], heart rate (HR) and respiration volume per time (RVT) were measured using pulse oximetry and a respiratory belt, respectively, and recorded with Spike2 (version 5.21, Cambridge Electronics Design Limited, Cambridge, UK).

### fMRI-NF setup

Acquired EPI data was submitted to real-time motion correction and spatial smoothing (FWHM 4 mm). For the localizer scan, real-time statistical analyses were carried out via an incremental general linear model (GLM) using Turbo-BrainVoyager (TBV) (Version 3.0, Brain Innovation, Maastricht, The Netherlands). Target ROIs in the respective groups were identified during a localizer scan based on the *t*-statistic of the contrasts of interest, which were defined as positive vs. neutral pictures in the NFE group and scene vs. face pictures in the NFS group. Target ROIs in the NFE group were limited to limbic and frontal portions of the anterior cerebrum based on models of emotion processing in the human brain [19]. This focus also helped to exclude areas involved in early visual processing. For the localizer scans of the NFE group, we used a previously described procedure [7] using pictures rated as positive, negative and neutral from the International Affective Pictures System (IAPS) [20]. We aimed for a control condition that would entail similar upregulation success and thus reward experience in both groups. Thus, the NFS group was presented with visual stimuli showing faces, scenes, and animals to localize higher visual brain areas. Patients in both groups were presented with four series of four pictures (1.5 s each) per category in pseudorandom order with alternating presentation and fixation baseline blocks. The parahippocampal place area (PPA) was chosen as the main target region in the NFS group. The PPA has been identified with the presentation of neutral scene stimuli [21, 22], but it is also activated during mental imagery of scenes [23], and can be upregulated with fMRI-NF [9].

The localizer scan was followed by six neurofeedback runs (100 volumes, first 6 volumes discarded to ensure T1 equilibrium magnetization), each containing four 20 s upregulation blocks alternating with 20 s rest blocks (the first rest block was 40 s long). Neurofeedback was provided with a visual thermometer display projected onto the screen in the scanner and provided continuously (i.e., updated with each volume) as described previously [7]. Real-time statistical analyses were carried using Turbo-BrainVoyager (TBV) (Version 3.0, Brain innovation, Maastricht, The Netherlands). The signal intensity of target areas was measured as the percent signal change (PSC) relative to baseline (see Suppl. Methods). Both groups were instructed to increase activation in their target areas during the upregulation periods and were informed that using imagery of positive stimuli (NFE) or imagery of scenes (NFS) might be a potential starting strategy. However, patients were not restricted in their mental strategies and could use any strategy that would enable them to achieve this. Moreover, patients were asked to practice successful mental imagery strategies at home between training sessions. For offline fMRI analyses, motion parameters and physiological measures (HR and RVT) were included as nuisance regressors in the General Linear Model (see Suppl. Methods).

### Statistical tools and analysis

The primary and secondary outcome measures for patients with complete data were analyzed using Statistical Package for Social Science (SPSS, version 23). To test for any significant differences between groups, linear regression analyses were performed on the outcome measures post-intervention (separately for the primary endpoint after session 5 and for the follow-up session) with pre-intervention scores as regressors of no interest. Further, the following regressors of no interest were entered to reflect the minimization procedure applied during randomization: gender, age, duration of illness, medication type, the score for the respective outcome variable at baseline, and HDRS-17 score at baseline. We deemed a Bonferroni-corrected *p* < 0.002 (*p* = 0.05/21 tests) to be appropriate for the secondary outcome measures. We also analyzed pre-post differences within group means. For all pre-post comparisons, % change and 95% confidence intervals of the changes in scores were calculated separately for both groups. Remission was defined by an HDRS-17 score ≤ 7, and remission rates were calculated based on all patients for whom we had complete data up to the relevant time point. Effect sizes (ES) of clinical outcome on the HDRS-17 were quantified using Hedges *g* based on difference scores with respect to baseline for both session 5 (post-intervention) and FU. Confidence intervals were bootstrapped based on 10,000 iterations using the R package bootES (version 1.3–20) [24]. Given the absence of group effects for HDRS (Results section), we also conducted post-hoc tests for evidence for a null effect (see Suppl. Methods and Suppl. Results). Obtained *t*-test values of ROI analyses were submitted to two-sided *t*-tests for each session to test for activation at group level. *p*-values were adjusted based on the false discovery rate (FDR) to corrected for multiple testing [25]. Given the absence of a group effect in upregulation and the similar clinical effects observed in both groups we also conducted equivalence tests based on participants’ median ROI *t* values across the four neurofeedback training sessions [26]. The test was conducted with a Welch *t*-test (corrected for unequal variances) and with a SESOI = −0.7 to 0.7 [with raw score lower limit 90% CI = −1.799 and upper limit 90% CI = 1.799].

### Clinical effects and self-efficacy

Self-efficacy describes an individual’s self-reported capacity to cope with challenges [27]. To test whether pre-post changes in self-efficacy (combined scores general and social self-efficacy score) predicted changes in depression scores, we carried out a regression analysis on residualized HDRS-17 scores of the primary endpoint (session 5). Specifically, to minimize potential confounding by baseline HDRS-17 and baseline self-efficacy scores, we regressed these in a first step from HDRS-17 scores at the primary endpoint. Obtained residuals were submitted to a robust (iteratively weighted least squares) regression analysis [28].

## Results

### Clinical outcomes

**Fig. 1:**
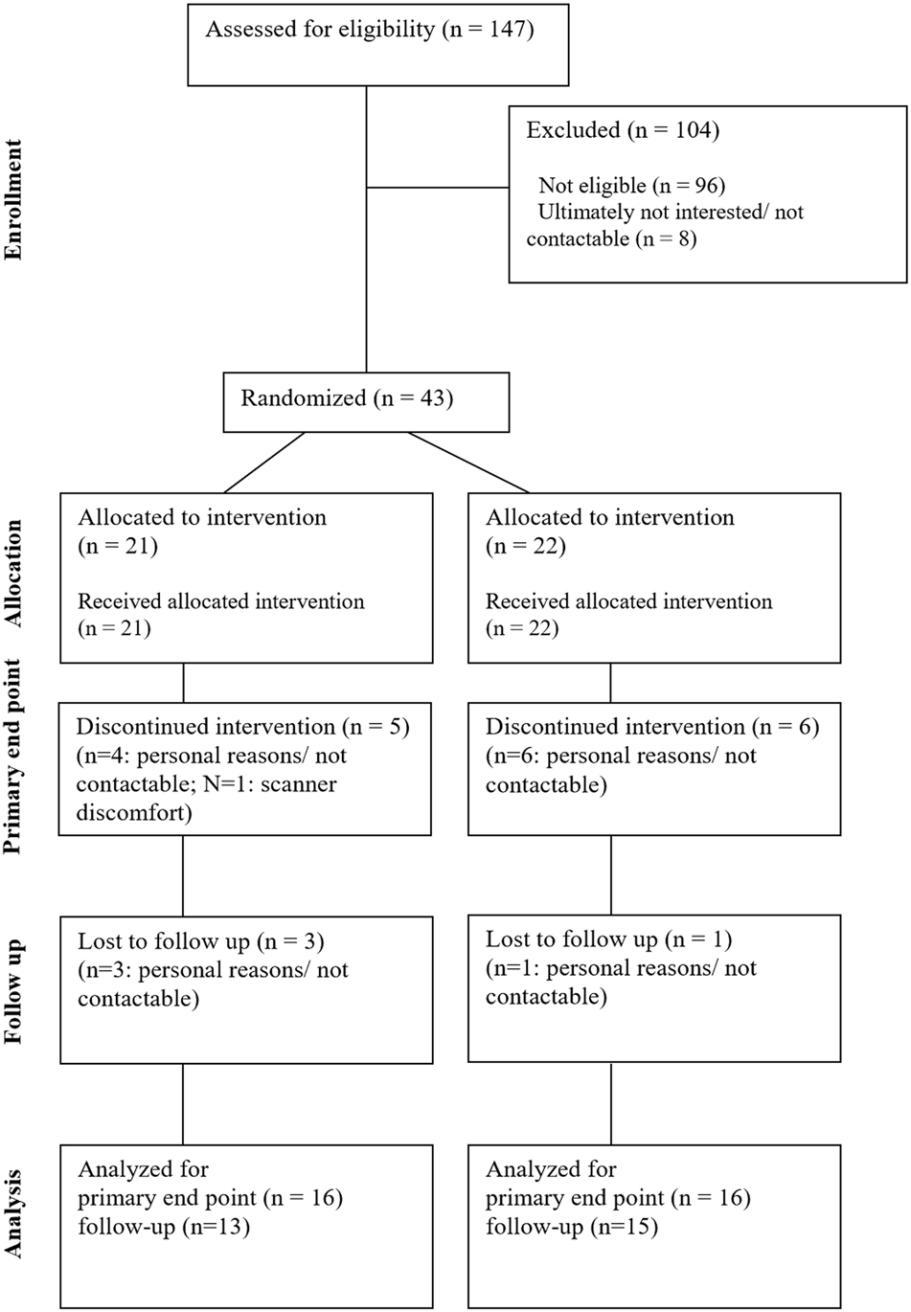
CONSORT diagram of clinical trial

The patient flow is summarized in the CONSORT diagram (Fig. 1). Recruitment ended in June 2014 when the target was achieved, and the last follow-up assessment was completed in September 2014. A total of 32 patients (16 in each group) completed the intervention. All 32 patients were included in the analysis of clinical, psychometric, and neuroimaging data at the primary endpoint. Further, 28 of 32 patients (88%) also attended follow-up and were included in the analysis of clinical and psychometric data at follow up. Randomization was successful such that there were no group differences at baseline in gender, age, handedness, HDRS-17, duration of depressive disorder or medication (Table 1). Second-rater scores for the HDRS-17 were available for 12 sessions, with high interrater correlation (Pearson’s correlation: *r* = 0.95, *p* < 0.001).

**Table 1:** Patient demographics and clinical baseline information. Combination of medication is

Characteristics	NFE Group (*n* = 16)	NFS group (*n* = 16)	Group differences	
			Statistical values	*p* values
Gender	Female = 11; Male = 5	Female = 10; Male = 6	Pearson *χ*^2^ = 0.139	0.71
Age	M: 47.19 years (SD:12.50)	M: 46.94 years (SD:12.74)	*t*_30_ = 0.6	0.96
Handedness	Right = 14; Left = 1; Ambidextrous = 1	Right = 13; Left = 3	Pearson *χ*^2^ = 2.037	0.36
Depression severity (HDRS-17)	19.88 (SD: 3.65)	19.09 (SD: 5.09)	*t*_30_ = 0.5	0.62
Duration of depressive disorder	19 years (SD:12.39)	18.56 years (SD:14.76)	*t*_30_ = 0.9	0.93
Medication	SSRI only = 4; Non-SSRI = 6; Combination = 6 (Combination of two antidepressants = 3; Augmentation = 3; Augmentation included either mood stabilizer, lithium, or 2nd generation antipsychotic in addition to antidepressant)	SSRI only = 7; Non-SSRI = 5; Combination = 4 (Combination of two antidepressants = 0; Augmentation = 4; Augmentation included either mood stabilizer, lithium, or 2nd generation antipsychotic in addition to antidepressant)	Pearson *χ*^2^ = 1.31	0.52

**Fig. 2:**
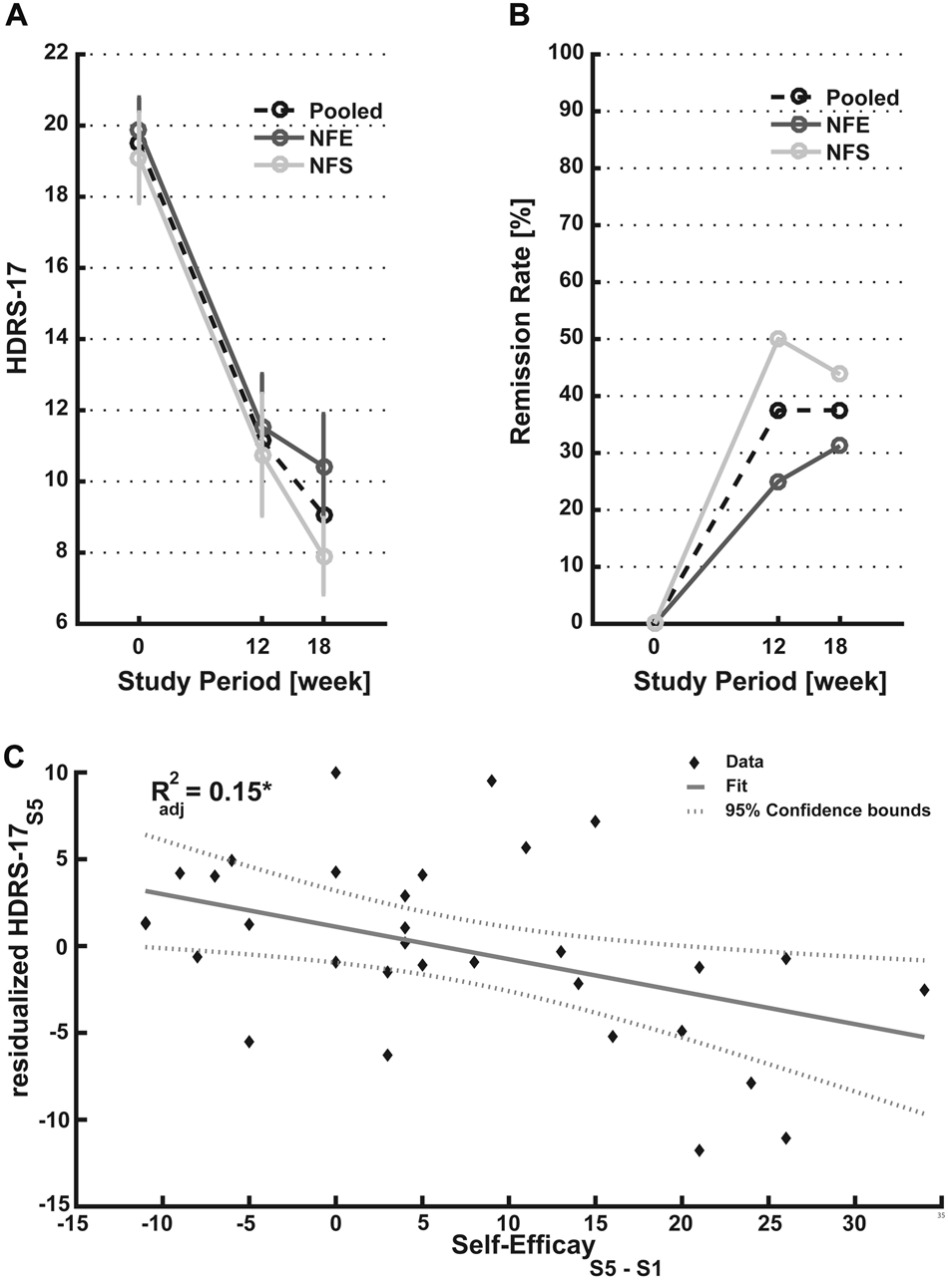
Main clinical outcome measure HDRS-17 for NFE and NFS groups at baseline, post-intervention, and follow up. **a** Mean and standard error of the mean (SEM) HDRS scores, **b** remission rate in %age based on HDRS, **c** Robust regression analysis between change in total self-efficacy scores, and residualized HDRS-17 scores at primary endpoint. *p < 0.05

There was no significant difference between groups on HDRS-17 (*B* = −0.415, [95% CI −4.847 to 4.016], *p* = 0.848) or any of the secondary outcome measures at the primary endpoint (see Table S1 and Suppl. Results section 2.3, including Bayesian analysis of covariance (ANCOVA) and equivalence tests). All pre-post comparisons of clinical and psychometric outcomes across the three sessions (baseline, end of intervention, and follow up) are documented in Table S2. Depressive symptoms in both groups improved similarly on the HDRS-17 (NFE: −8.34 [95% CI −4.92 to −11.77]; NFS:−8.34 [95% CI −5.81 to −10.87]; Fig. 2a) and secondary outcome measures. The NFE group improved by 42% on the HDRS-17, the NFS improved by 44%. Overall, the effect size for HDRS-17 was *g* = 1.46 [95% CI 0.97 to 1.95] at session 5 (NFE *g* = 1.23; [95% CI 0.63 to 1.92] vs. NFS *g* = 1.67 [95% CI 0.94 to 2.48]) and depression scores decreased by about 43% [95% CI 24 to 64%] across groups.

Figure 2b shows the remission rates, which was overall 37.5% [95% CI 22.9 to 54.8%; 12/32 patients] with 25% for NFE (4/16 patients) and 50% for NFS (8/16 patients). There was no significant difference between groups on the HDRS-17 (*B* = −1.121 [95% CI −4.948 to 2.706], *p* = 0.548) or any of the secondary clinical outcome measures at follow-up (Table S1). Both groups improved compared to baseline on the HDRS-17 (NFE: −9.65 [95% CI −6.25 to −13.06]; NFS: −11.53 [95% CI −8.61 to −14.45]) and the secondary outcome measures. At FU, the NFE group had improved by 48% on the HDRS-17 compared to baseline, and the NFS group had improved by 59%. The overall effect size for HDRS-17 improvement at FU was *g* = 1.88 [95% CI 1.24 to 2.54]; (NFE: *g* = 1.57 [95% CI 1.04 to 2.14] vs. NFS: *g* = 2.05 [95% CI 0.96 to 3.72]). Figure 2b shows the overall remission rate remained at 37.5% (NFE = 31%; NFS = 44%). Group differences of outcomes of other psychological measures were largely not significant and those that were did not survive correcting for multiple comparison (see Suppl. Results).

Given the absence of a group mean difference on the HDRS-17, but an overall strong and similar clinical response in both groups, we tested whether unspecific effects reported for high-technology augmentation therapy could be rejected. For example, an improvement of 5.5 points on the HDRS-17 was reported for sham whole-body hyperthermia (WBH) treatment [17]. Based on the complete sample (*N* = 32) we carried out a one-sample two-sided t-test against a test value of 6 found that our effect was significantly larger than that expected placebo response (*t*_31_ = 2.385, *p* = 0.023, Cohen’s *d* = 0.422 [95% CI 0.057 to 0.780]).

### Self-efficacy and clinical improvement

The exploratory robust regression analysis suggested that changes in self-efficacy predicted residualized depression scores at the primary endpoint (*R*^2^ = 0.18, adjusted *R*^2^ = 0.15, *β* = −0.187 ± 0.073, Fig. 2c), such that increase in self-efficacy was associated with less depression severity (*t*_30_ = −2.551, *p* = 0.016).

### Region of interest analysis

**Fig. 3:**
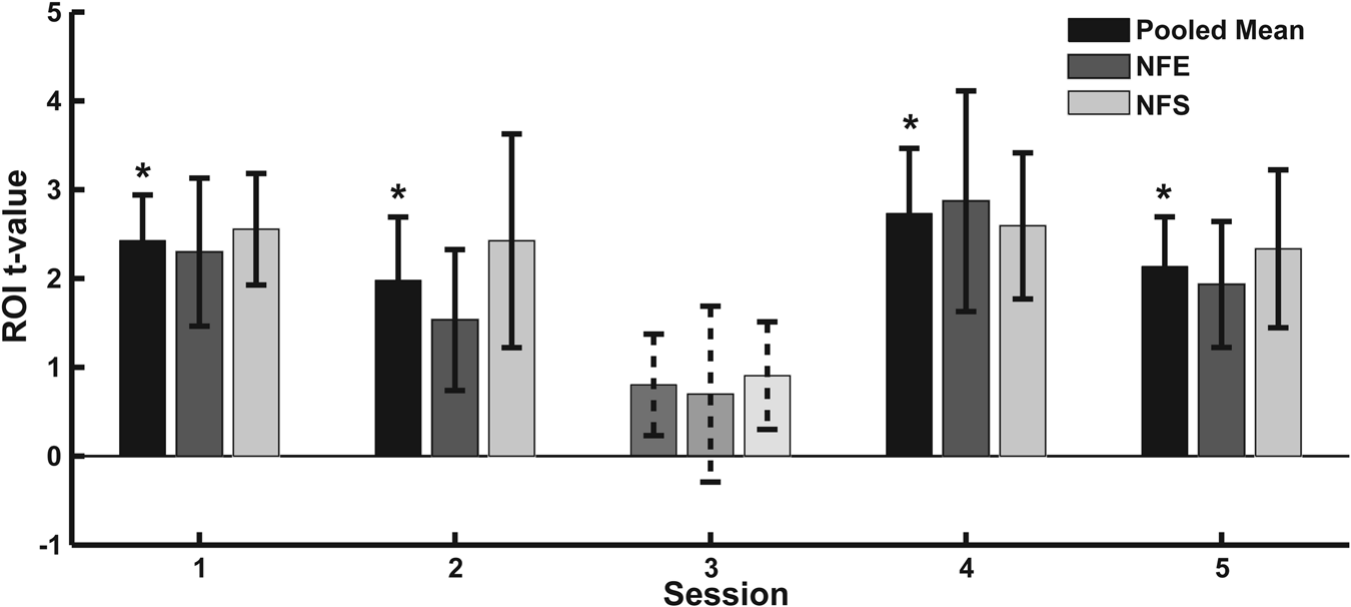
ROI analysis. Average *t*-value of target ROIs for each session. Pooled mean shown, as well as means for both groups with error bars showing standard error of the mean (SEM). *p < 0.05 at FDR correction

Patients from both groups received neurofeedback training from target ROIs of similar size (measured in number of mm^3^ voxels in Talairach space; NFE = 2190 ± 293 vs NFS = 2913 ± 342, *t*_158_ = −1.606, *p* = 0.110). Patients upregulated the target ROIs during all neurofeedback sessions (session 1: *t*_31_ = 4.723; *p*_FDR_ < 0.002, session 2: *t*_31_ = 2.772, *p*_FDR_ = 0.011; session 4: *t*_31_ = 3.726, *p*_FDR_ = 0.002; session 5: *t*_31_ = 3.809, *p*_FDR_ = 0.002; Fig. 3), but not during the transfer session (session 3: *t*_31_ = 1.404; *p*_FDR_ = 0.170). The difference between the average upregulation across neurofeedback sessions and the transfer session was significant (*t*_31_ = 2.397, *p* = 0.023). There was no evidence for an effect of group (*F*_1,30_ = 0.12, *p* = 0.73), session (*F*_4,120_ = 1.965, *p* = 0.104), or group × time interaction (*F*_4,120_ = 0.160, *p* = 0.958). Further, an equivalence test suggested that both groups activated ROIs to a similar extent (*t*_29.8_ = 1.702, *p* = 0.049, raw score lower limit 90% CI = −1.795 and upper limit 90% CI = 1.290).

### Whole-brain analysis

**Fig. 4:**
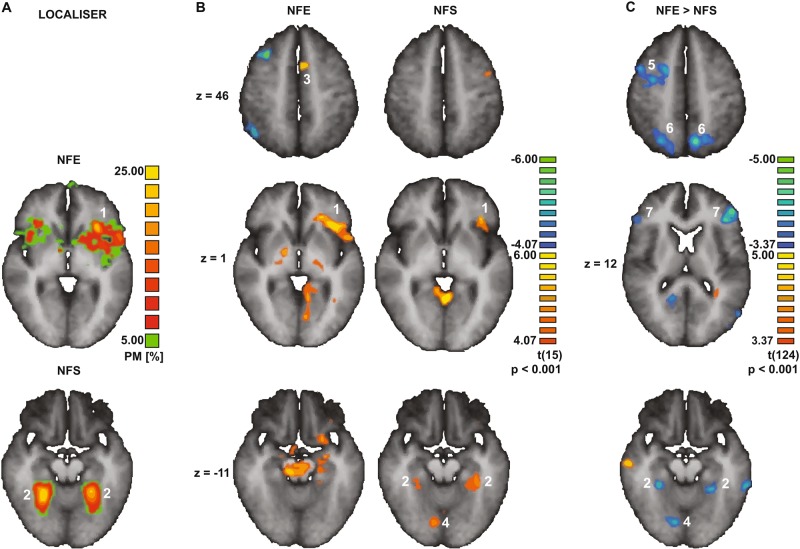
Whole-brain analysis. **a** Probability map (PM) of the localizer. **b** Activity of intervention groups, shown separately for NF-emotion and NF-scene group. **c** Group contrast. Key areas are labeled with numbers: (1) Insular cortex/ ventrolateral prefrontal cortex, (2) parahippocampal Place area (PPA), (3) supplementary motor area, (4) lingual gyrus, (5) premotor cortex, (6) superior parietal lobule, and (7) ventrolateral prefrontal cortex. Statistical maps cluster-threshold corrected for multiple comparison (*p* < 0.001)

Figure 4a shows the distribution of the main locus for target region locations of the NFE and NFS groups (but see also Fig. S1 for more details). Patients in the NFE group were primarily trained on anterior brain areas (e.g., insular and striatum), patients in the NFS group mainly trained on the PPA. Figure 4b shows brain regions involved in the neurofeedback training (contrast regulate > rest). For the NFE group, limbic and subcortical regions (e.g., the insula, caudate, and hippocampus) were activated while the dorsolateral prefrontal cortex was deactivated (Fig. 4b; Table S3). For the NFS group, mainly higher visual areas were activated including the bilateral PPA as the main target region of this group (Table S3). A direct group comparison (contrast NFE > NFS) suggested that the NFS group showed more activation of the PPA and frontal regions (Fig. 4c and Table S4).

## Discussion

We demonstrate feasibility and clinical efficacy of fMRI neurofeedback training in medicated patients with a longstanding history of clinical depression. Neurofeedback training of emotional areas was not superior to the control neurofeedback intervention. Nonetheless, both intervention groups showed a clinically significant improvement of the primary outcome measure with over 40% reduction in the HDRS-17. The overall remission rate was about 38%. Importantly, both measures were maintained, if not slightly improved at follow-up (Fig. 2a, b). Further, both groups improved to a similar extent on the secondary clinical outcome measures of anxiety and depression and reported positive effects on their quality of life. Taken together, this trial reproduced and extended findings from a recent placebo-controlled fMRI-NF RCT in unmedicated patients [8].

The whole-brain analysis of the NFE group showed activation of the insula and the ventral striatum, indicating that patients engaged in emotion self-regulation. This is encouraging because striatal areas and the insula have been linked to positive reappraisal of emotions [29]. Both emotion self-regulation and reappraisal constitute important elements in psychotherapeutic treatment for depression; however, they can be impaired in depressed patients [30]. Our data suggest that fMRI-NF training could indeed help patients in training this capacity. Of interest, activated voxels in the NFS and NFE partly overlapped, mainly in the anterior insula (Fig. 4b and Table S3). The (anterior) insula has been implicated in various cognitive tasks that may explain this overlap, including its involvement in the integration of visual information that is required for mental imagery of scenes [31], self-regulation of affective as well as executive functions [32, 33], and online monitoring of performance [34], a factor for which groups were matched. Without more fine-grained fMRI measurements, however, we cannot determine if the overlapping clusters between groups reflect activation of the same neuronal populations. On a more general level, though, this overlap highlights the difficulty of selecting strictly separated target networks for different neurofeedback conditions.

A key question is why the active control neurofeedback group (NFS) also showed a substantial clinical improvement. The improvement of the NFS group may be explained by a similarly rewarding experience and the type of mental imagery training patients engaged in. Both groups upregulated target ROIs to a similar extent, as shown by equivalence tests. Hence, also participants in the NFS group experienced success of self-regulation and they were positively reinforced for task success. Generic psychological or physiological factors could thus be important for any clinical efficacy of neurofeedback training in depression. We have earlier suggested that self-regulation experience during neurofeedback training may be associated with an increase of self-efficacy and explain therapeutic effects of fMRI-NF training in depression [7, 35]. This conjecture is supported by an exploratory robust regression analysis between changes in self-efficacy and residualized HDRS-17 scores (Fig. 2c). Self-efficacy has been shown to mediate vulnerability to develop depressive symptoms [36], as well as the effect of depression on developing detrimental behavior including nicotine abuse [37]. The reported changes in self-efficacy may thus yield secondary benefits including less vulnerability to relapse and cognitive resources to cope with challenges [27]. However, larger trials of longer duration that include transfer task that engage similar processes are needed to test whether self-efficacy may represent a psychological mechanism underlying therapeutic effects of fMRI-NF training [38]. We further note that the task of the NFS group—imagining relaxing visual scenes—may also have had a therapeutic effect. Relaxation therapies are used in clinical practice to treat depression [39, 40]. Taken together, our active control group likely received therapeutic self-regulation training and thus showed substantial improvement. It is hence possible that our control intervention was too conservative with respect to our aim of showing superiority of targeting the affective brain.

Two (partly related) remaining questions are whether the reported clinical findings are specific, and whether they are comparable to effective active interventions or usual care. As the current design did not include a sham-feedback or a care as usual control group, we will argue based on epidemiological data that the clinical effects reported here (1) exceed spontaneous remission rates, (2) are comparable to clinical effects reported for first line treatment as well as current best augmentation treatment alternatives, and (3) exceed placebo effects reported for sham-controlled high-technology therapies

First, the improvement of 40% on the HDRS-17 exceeds the minimal clinically relevant improvement of about 27% [41] and thus represents a substantial clinical effect. Findings also exceed expected regression to the mean effects for chronically depressed and partly treatment-resistant patients (the average duration of illness was 19 years), for which epidemiological data only suggests a rather sluggish improvement of 10–15% [42]. Further, given that patients in our trial had received stable medication for at least three months, the main response to pharmacological treatment had likely already occurred before enrollment [43].

Second, the effects reported here across both groups for the primary endpoint—about 8.3 points improvement and 37.5% [95% CI 22.9 to 54.8] remission on the HDRS-17—are comparable to initial responses found for first line pharmacological treatment (8 to 9 points improvement on the HDRS-17 and 36.8% remission) in patients with comparable depression severity and chronicity [43, 44]. Indeed, the confidence interval around our remission rate excludes the estimated remission rate (22%) for placebo groups in first level pharmacotherapy treatment [45]. However, these comparisons seem rather conservative given that patients received fMRI-NF as an augmentation, or even as a second augmentation treatment (one third of patients were already receiving combined pharmacological treatment), because augmentation treatments usually yield gradually less clinical improvement. Noteworthy, the presented clinical effects are similar and partly larger than effects reported for other accepted augmentation strategies: the “Sequenced Treatment Alternatives to Relieve Depression” (STAR*D) trial reported on average 35% remission for first augmentation, with about 36% for non-SSRIs and 29.4% for behavioral therapy, and remission rates of only 20.5% for secondary augmentation therapy [43].

Third, our clinical findings are also comparable to reported effects of other high-tech interventions for depression. We note that the size of non-specific treatment effects depends on patients’ expectancies, beliefs, as well as the psychosocial context [46], and non-specific treatment effects may thus be particularly large for fMRI-NF. However, a recent sham-controlled RCT of fMRI-NF of similar size in (unmedicated) depressed patients only found a marginal improvement of 2 points (HDRS-21) and 6% remission in a sham control group [8]. In contrast, their treatment group that engaged in similar fMRI-NF training as our NFE group improved by about 11.5 points and showed 32% remission. Likewise, our results are comparable for treatment groups and outperform placebo groups in other sham-controlled high-technology interventions tested for depression: meta-analyses of transcranial magnetic stimulation (TMS) trials suggest about 35% remission for treatment groups, while sham TMS groups show only 5–10% remission [47, 48]. Further, one whole-body hyperthermia (WBH) trial reported an improvement of 8.3 points (HDRS-17) and a remission rate of 40% remission for their treatment group, while WBH sham only led to a placebo response of 5.5 points and 0% remission [17]. Importantly, we could reject a large placebo response of six points in an exploratory analysis, thereby showing superiority of our clinical findings. Altogether, this survey of the size of expected placebo responses suggests that the clinical effects found in our trial exceed a mere regression to the mean or placebo effect. Rather, our findings are comparable to treatment effects reported for accepted augmentation therapies and other high-technology therapies currently investigated in depression.

Another high-technology intervention tested in depression is electroencephalography neurofeedback (EEG-NF). We have reviewed the available literature earlier where we found that it is limited to a small number of studies [35]. One randomized single-blind EEG-NF trial observed improvements similar to those as reported here (7.25 points improvement on the HDRS) after 5 weeks EEG-NF training [49], although we note that patients in this trial were less severely depressed and partly already remitted at baseline. An even larger improvement (from 21.38 to 6.23 on the HDRS-17) was reported in a more recent 8-week open-label (i.e., unblinded) study of EEG-NF [50]. However, both studies lacked active self-regulation control groups and thus do not allow estimating the expected placebo effect of EEG-NF in depression.

To test directly for a superiority of fMRI-NF over treatment-as-usual and lower cost alternatives, future trials with larger sample sizes could for instance pit neurofeedback against a non-MRI based control intervention such as biofeedback, fMRI-informed EEG-NF [51], or relaxation groups. Such a trial design would address some of the non-specific effects that may play a role such as exposure to technology, general self-regulation training, enhancement of self-efficacy and reflection on bodily states. However, it is important for us to consider the difference between trying to identify the specific effects of fMRI-NF and trying to ascertain if it is a treatment which is relatively more or less effective than other active interventions or usual care. These will naturally lead to different comparators in future studies [52].

In conclusion, we found no evidence for clinical superiority of the self-regulation training of emotion areas over that of higher visual areas. Both groups showed a clinical improvement that was comparable to effects reported for treatment as usual and exceeded expected regression to the mean and commonly observed placebo effects. Hence, this trial demonstrated that if moderately to severely depressed patients can upregulate a brain area that is not immediately linked to affective processing (NFS group), they can experience similar clinical benefits compared to patients who completed emotion-focused neurofeedback training (NFE group).We suggest that the experience of brain control and the positive reinforcement of mental imagery may be necessary components for therapeutic effects of neurofeedback and should therefore be considered for future designs. Overall, neurofeedback was well tolerated, but we would suggest that any clinical neurofeedback protocols should be clinically supervised and entail monitoring of mental strategies and psychological effects, so that patients can discuss their individual experiences and protocols can be adjusted if needed. We suggest that further exploration of the clinical efficacy of real-time fMRI neurofeedback protocols and neural as well as psychological mechanisms is needed.

## Electronic supplementary material

Supplemental Material

Supplementary figure 1
