# Supplementary material for: Targeting the affective brain—a randomized controlled trial of real-time fMRI neurofeedback in patients with depression
Source: Neuropsychopharmacology. 2018 Jun 23;43(13):2578–85. doi: 10.1038/s41386-018-0126-5 (PMC6186421; doi:10.1038/s41386-018-0126-5)
Supplement: Supplementary file 1 — Supplemental Material [file 41386_2018_126_MOESM1_ESM.docx]

**SUPPLEMENTARY MATERIAL**

**Supplementary Methods**

- 1. **Real-time fMRI setup**

Both groups were instructed to increase activation in their target areas during the upregulation periods. Patients were not restricted in their mental strategies and could use any strategy that would enable them to achieve this. They did not receive specific instructions, however, as a starting strategy they were advised to imagine visual stimuli similar to those seen in the localizer. During rest periods patients were instructed to engage in simple mental calculations (counting backwards from 99 in steps of 3). Patients were made aware of the temporal delay (6s) associated with the blood oxygen level dependent (BOLD) between changes in mental imagery and visual feedback on the thermometer.

Neurofeedback was provided based on the Percent Signal Change of the target ROI and presented using PsychoPy (Peirce, 2007). Specifically, the feedback was calculated as the running average of the preprocessed BOLD signal over the three preceding TRs, normalized by the mean BOLD signal of the last five TRs from the preceding baseline block. The maximum feedback signal was set to 1 percent signal change, the thermometer contained ten bars such that each bar represented 0.1 percent signal change. The thermometer was continuously updated and shown during the neurofeedback and rest period. During the transfer session (session 3) no feedback was provided and patients were instead presented with a static thermometer display.

- 1. **Homework**

Patients were provided with a CD-ROM that replayed the neurofeedback screens used in the scanner (but with static thermometers) in order to give them the opportunity to practice their mental imagery strategies at home, for two 20-minute sessions in the intervals between scanning sessions 1-4, and for four sessions between scanning sessions 4 and 5. They were asked to document their experience and successful strategies used for target ROI upregulation in a diary (not reported here).

- 1. **Additional measures (not reported here)**

Patients’ employment status and healthy service utilization was also recorded. Furthermore, patients completed the Profile of Mood States questionnaire before and after every neurofeedback session to assess the current mood state and level of fatigue.

- 1. **Offline fMRI analysis**

The neuroimaging data was analyzed offline using Brain Voyager QX 2.8.4 (Brain Innovation, Maastricht, the Netherlands) and custom-written MATLAB scripts (Version 8.2, The MathWorks Inc., Natick, MA, 2000). T1 images were inhomogeneity corrected and normalized to Talairach space. To correct for head movements, spatial alignment of all volumes to the first volume of the localizer was performed. The fMRI data was co-registered to T1 images based on anatomical landmark points, and co-registration was manually corrected if necessary. The data was normalized to Talairach space using rigid body transformation and scaling. Linear drifts were removed and a temporal high-pass filter (2 cycles) was applied to remove non-linear drifts in the time series. Data were spatially smoothed using a Gaussian kernel with 6-mm full width at half maximum and temporally smoothed with a Gaussian kernel with 3s full width at half maximum. Motion parameters and physiological measures (heart rate, respiration volume per time) were z-transformed and included as nuisance regressors in the General Linear Model. To quantify the homogeneity of target areas within and distinctness of target areas between the groups probability maps were calculated based on the Talairach coordinates of chosen target areas. To test whether patients successfully upregulated target areas within a session, ROI analyses were performed for each session. T-values of patient’s target regions were submitted to an ANOVA with the factors intervention (NFE vs. NFS) and session (five levels).

To identify task specific brain activity patterns, whole brain analyses were computed offline for both groups separately using a random effect General Linear Model and a task versus baseline contrast was defined. To test for effects of time, group and an interaction, an ANCOVA random effect General Linear Model was calculated and contrasts for intervention group (NFE vs. NFS) and time (linear contrast over all five sessions) tested. To limit multiple comparison correction, a mask was used that covered the cerebrum and upper brain stem and upper cerebellum. Active brain regions were identified based on the nearest gray matter coordinates using the Talairach Daemon atlas tool (Lancaster *et al*, 2000). The statistical parametric maps were multiple comparison-corrected using the Monte Carlo simulation implemented in Brain Voyager (primary threshold p < 0.001; cluster-extent threshold p < 0.05) (Eklund *et al*, 2016). For the between-group contrast this primary threshold corresponded to FDR correction (p < 0.05).

- 1. **Bayesian analysis of covariance (ANCOVA) and equivalence tests**

Given non-significant group comparisons, we conducted post-hoc tests to see if 1) evidence for a null effect based on an assumed effect size used for the power calculation could be found (Bayesian Linear Regression) and 2) a smallest clinically meaningful standardized effect size could be rejected (equivalence test). Bayesian ANCOVA with treatment group as a fixed effect and the randomization factors (gender, age, duration of illness, medication type and the HDRS-17 score at baseline) as nuisance covariates were carried out using JASP (JASP Team (2016), version 0.8.0.1). The prior for group as a fixed effect was scaled by r = 1.2, which reflects the effect size used for the power analysis. The prior for covariates was scaled by a default value (r = 0.354). To test if the smallest effect size of interest (SESOI) could be rejected, two one-sided tests for equivalence (TOST) were performed (Schuirmann, 1987) using the TOSTER package (version 0.2.3) in RStudio (version 1.0.136). A SESOI = -0.3 to 0.3 [with raw score lower limit 90% CI = -1.695 and upper limit 90% CI = 1.695] was chosen, which has been suggested as a common effect size for the additional benefit of anti-depressive medication compared to placebo (Kirsch *et al*, 2008). Although this was lower than our originally expected effect size, this analysis allows test if we can confidently rule out group effects that are clinically still relevant. Moreover, based on the confidence interval around the empirical effect size for a group difference we calculated the equivalence bound (i.e. the lower and upper bound for the smallest SESOI that could be rejected in either direction). This allowed us to explore the limit of effect sizes that one could reject based on the data from the current sample.

**1.6. Intention to treat analysis**

In the current design intention to treat analyses were limited to follow-up tests of baseline characteristics because clinical re-assessment of the primary outcome measure (as well as all other clinical measures) was performed after trial completion (session 5). With the available in the present trial we tested 1) whether attrition rates differed between treatment conditions (using frequentist and Bayesian binominal tests), and 2) whether depression characteristics (severity and duration) were associated with higher drop out using a frequentist ANCOVA, as well as frequentist and Bayesian t-tests. For the ANCOVA, baseline HDRS-17 or duration were respectively entered as the dependent variable. Binary coded completion status was entered as a fixed factor, and age, gender, dummy-coded medication type and group allocation were entered as covariates. Both baseline characteristics (HDRS-17 and duration of illness) were further compared between completers and non-completers using a frequentist Welch t-test (Delacre *et al*, 2017) and a Bayesian independent t-test with a normal prior distribution (N(0, 0.3)) that was scaled by what is considered minimal clinically important difference (Kirsch *et al*, 2008).

**Supplementary Results**

**2.1. Attrition, Termination and Demographics**

The patient flow is summarized in the CONSORT diagram (Figure 1). A total of 147 patients agreed to be screened for the present study, 104 patients were excluded because they did not meet eligibility criteria or because they decided not to participate in the trial. The remaining 43 patients were randomized. Recruitment ended in June 2014 when the sample size target was achieved, and the last follow-up assessment was completed in September 2014. 32 patients (16 per group) completed the intervention, their demographical data are documented in Table 1. 28 patients (88%) also participated in the follow up assessment. With respect to the total number of randomized patients, the attrition rate across both groups was ~26% at the end of the intervention (session 5) and ~35% at follow up. Three patients in the NFE group increased their antidepressant medication during the trial, and five patients (four in the NFE and one in the NFS group) decreased or stopped medication. Hence, overall attrition rates in our trial were relatively low and comparable to other non-invasive intervention methods tested for MDD (Hollon *et al*, 2014; Janssen *et al*, 2016), suggesting that the procedure was well accepted by patients.

**2.2. Homework completion and experience and reported side effects.**

We asked participants to record their experience both during the scanning sessions and the homework in their diaries. We analyzed the 24 diaries that were handed in after the completion of the study. The 11 patients in the NFE group who completed the diary did on average 8.36 out of the 10 possible homework session between scanning session 1 and 5 (range: 4-10) and the 13 patients in the NFS group did on average 8.76 sessions (range 5-10).

No major adverse effects were reported. Three patients in each group reported frustration with some homework sessions, mainly because of difficulty focusing and concentrating. One patient in the NFE group reported feeling agitated after one homework session. Another patient in the NFE group reported an initial positive response to the homework session, followed by a rebound in low mood after a few days. Five patients in the NFS group and one in the NFE group reported exhaustion or fatigue after scanning, whilst two patients in the NFE and three in the NFS group reported transient headaches or other aches or dizziness. One patient in the NFE group reported transient ear ringing after one scanning session. Fatigue, backache, ear ringing and dizziness are commonly reported non-specific effects of lying in an MRI scanner.

**2.3. Bayesian ANCOVA and Equivalence testing**

When controlling for randomization factors, the Bayesian analysis found moderate evidence for a null effect of group at both the primary endpoint (BF01 = 6.01) and at follow-up (BF01 = 3.51). Equivalence tests were undetermined at both the primary endpoint (t_27.62_ = -0.849; p = 0.202, raw score lower limit 90% CI = -3.40 and upper limit 90% CI = 3.40) and at follow-up (t_24.68_ = - 0.104; p = 0.541, raw score lower limit 90% CI = -5.478 and upper limit 90% CI = 1.718), indicating that the current sample was too small to provide evidence for the lack of an effect within the range of the SESOI. Lastly, based on the confidence intervals of the empirical effect size we determined that only effect sizes larger than Cohen’s = |0.6| in either direction could have been rejected in the current sample. Taken together, the Bayesian analysis whose prior was scaled based on the effect size from the original power calculation suggested evidence for the absence of a group effect. In contrast, equivalence tests could not rule out group differences of the size that have been described for add-on treatment, but only effects that were at least twice as large.

**2.4. Intention to treat analysis**

Among non-completers, group allocation was balanced with a proportion of five patients from NFE to 6 patients from the NFS group (0.455 vs. 0.545, p =1; BF_01_ = 2.707). The ANCOVA found no evidence that completion status explained baseline HDRS-17 scores (F_1, 35_ = 0.361, p = 0.552) or duration of illness (F_1, 35_ = 0.467, p = 0.499). Likewise, a frequentist independent t-test found no significant difference in baseline HDRS-17 between completers and non-completers (t_14.72_ = -0.486, p = 0.634, Cohen’s d = -0.178), while a Bayes Factor remained inconclusive (BF_10_ = 0.808). Similarly, no significant difference was found for duration of illness (t_28.82_ = -0.302, p = 0.765, Cohen’s d = -0.093) and Bayes Factor remained inconclusive (BF_10_ = 0.768). Taken together, intention to treat analyses suggested no significant differences between completers and non-completers with regards to group allocation, or key baseline characteristics. Bayesian test results only provided anecdotal evidence for the absence biases.

**2.5. Target regions selected during localizer**

Figure 3A shows the main target regions selected during the visual localizer. To provide a more detailed overview, Figure 1S shows selected slices also in a sagittal and coronary view.

**2.6. Other Psychological Measures**

Table S1 documents the changes in the other psychological and quality-of-life measures for both groups. Table S2 documents group comparisons. There were no significant group differences except for SSE at S5 (B = 3.350 [95% CI 0.408 to 6.302], p = 0.027) and at FU (B = 4.206 [95% CI 0.410 to 8.001], p = 0.032), as well as BAS at FU (B = 5.763 [95% CI 0.207 to 11.043], p = 0.043), which all did not survive Bonferroni correction for multiple testing.

**Table S1.** Estimated coefficients (B) and standard errors (SE) for treatment-by-time interactions from fitting a linear regression on the outcome measures with randomization and baseline scores as regressors of non-interest for primary and secondary clinical and

psychometric measures at primary endpoint and follow-up.

| **Outcome measure** | **B** | **SE** | **t** | **95% CI** | **p value** |
| --- | --- | --- | --- | --- | --- |
| **Primary endpoint (session 5)** | | | | | |
| Hamilton Depression Rating Scale | -0.415 | 2.147 | -0.193 | -4.847 to 4.016 | 0.848 |
| Hospital Anxiety and Depression Scale (Anxiety Subscale) | -0.997 | 1.124 | -0.887 | -3.322 to 1.328 | 0.384 |
| Hospital Anxiety and Depression Scale (Depression Subscale) | -0.860 | 1.631 | -0.527 | -4.235 to 2.515 | 0.603 |
| Behavioral Inhibition System | 0.852 | 0.858 | 0.993 | -0.924 to 2.627 | 0.331 |
| Behavioral Activation System | 3.091 | 1.769 | 1.747 | -0.569 to 6.751 | 0.094 |
| Quality of Life Scale | 1.510 | 3.438 | 0.439 | -5.603 to 8.623 | 0.665 |
| Thought Control Questionnaire | -2.617 | 3.301 | -0.793 | -9.447 to 4.212 | 0.436 |
| Thought Control Ability Questionnaire | -5.532 | 4.515 | -1.225 | -14.871 to 3.807 | 0.233 |
| Self-Efficacy Scale. General Self-Efficacy Subscale | 3.350 | 3.344 | 1.002 | -3.568 to 10.268 | 0.327 |
| Self-Efficacy Scale. Social Self-Efficacy Subscale | 3.355 | 1.425 | 2.355 | 0.408 to 6.302 | 0.027 |
| EuroQol research foundation questionnaire | 0.985 | 0.953 | -1.034 | -2.988 to 1.017 | 0.315 |

| **Follow-up** | | | | | |
| --- | --- | --- | --- | --- | --- |
| Hamilton Depression Rating Scale | -1.121 | 1.835 | -0.611 | -4.948 to 2.706 | 0.548 |
| Hospital Anxiety and Depression Scale (Anxiety Subscale) | -0.006 | 1.50 | -0.004 | -3.145 to 3.134 | 0.997 |
| Hospital Anxiety and Depression Scale (Depression Subscale) | -0.865 | 1.843 | -0.485 | -2.963 to 4.753 | 0.633 |
| Behavioral Inhibition System | 0.219 | 1.354 | 0.162 | -2.615 to 3.053 | 0.873 |
| Behavioral Activation System | 5.625 | 2.589 | 2.173 | 0.207 to 11.043 | 0.043 |
| Quality of Life Scale | 2.635 | 4.0 | 0.659 | -5.737 to 11.007 | 0.518 |
| Thought Control Questionnaire | -4.273 | 4.051 | -1.055 | -12.752 to 4.207 | 0.305 |
| Thought Control Ability Questionnaire | -1.396 | 4.750 | -0.294 | -11.337 to 8.545 | 0.772 |
| Self-Efficacy Scale. General Self-Efficacy Subscale | 5.763 | 3.656 | 1.576 | -1.888 to 13.415 | 0.131 |
| Self-Efficacy Scale. Social Self-Efficacy Subscale | 4.206 | 1.814 | 2.319 | 0.410 to 8.001 | 0.032 |
| EuroQol research foundation questionnaire | -0.251 | 0.678 | -0.370 | -1.697 to 1.195 | 0.717 |

**Table S2.** Pre-post change for each group results for primary and secondary clinical scored and psychometric measures at primary endpoint and follow-up. Reductions on the HDRS, Hospital Anxiety and Depression Scale and EuroQol research foundation questionnaire denote clinical/ functional improvement.

| Outcome Measures | Change from baseline to primary endpoint (12 weeks)  for NFE / NFS | | | | | | | | |
| --- | --- | --- | --- | --- | --- | --- | --- | --- | --- |
|  | M | 95% CI | | % -age | | Hedge’s g | | 95% CI | |
| Hamilton Depression Rating Scale | -8.35/  -8.34 | -4.92 to -11.77/  -5.81 to -10.87 | | -42/  -44 | | -1.23 /  -1.67 | | -1.92 to -0.63/  -2.48 to -0.94 | |
| Hospital Anxiety and Depression Scale (Anxiety Subscale) | -1.81/  -3.19 | 0.07 to -3.69/  -1.44 to -4.93 | | -14/  -25 | | -0.49 /  -0.92 | | -.97 to 0.09/  -1.26 to -0.53 | |
| Hospital Anxiety and Depression Scale (Depression Subscale) | -3.06/  -3.81 | -0.87 to -5.25/  -1.35 to -6.28 | | -23/  -31 | | -0.71 /  -0.78 | | -1.09 to -0.24/  -1.71 to -0.01 | |
| Behavioral Inhibition System | 0.75/  0.94 | -1.23 to 2.73/  -0.06 to 1.94 | | 6/6 | | 0.19 /  0.47 | | -0.33 to 0.61/  -0.04 to 0.95 | |
| Behavioral Activation System | -2.69/  0.94 | -5.71 to 0.33/  -1.09 to 2.96 | | -8/3 | | -0.45 /  0.23 | | -1.11 to 0.10/  -0.03 to 0.95 | |
| Quality of Life Scale | 7.63/  8.94 | 2.7 to 12.55/  3.83 to 14.05 | | 14/16 | | 0.78 /  0.88 | | 0.31 to 1.24/  0.25 to 1.54 | |
| Thought Control Questionnaire | 5.25/  2.19 | -0.5 to 11/  -1.85 to 6.23 | | 9/4 | | 0.46 /  0.27 | | -0.07 to 0.79/  -0.22 to 0.74 | |
| Thought Control Ability Questionnaire | 9.19/  3.75 | 1.58 to 16.79/  -3.03 to 10.53 | | 17/7 | | 0.61 /  0.28 | | 0.04 to 1.15/  -0.25 to 0.86 | |
| Self-Efficacy Scale, General Self-Efficacy Subscale | 3.56/  5.81 | -1.53 to 8.66/  0.61 to 11.02 | | 8/12 | | 0.35/  0.56 | | -0.13 to 0.86/  0.13 to 1.05 | |
| Self-Efficacy Scale, Social Self-Efficacy Subscale | 0.69/  3.94 | -1.29 to 2.67/  1.86 to 6.01 | | 4/28 | | 0.18/  0.96 | | -0.33 to 0.64/  0.25 to 1.64 | |
| EuroQol research foundation questionnaire | -0.38/  -1.00 | -2.25 to 1.48/  -2.18 to 0.18 | | -3/-10 | | -0.21/  -0.38 | | -0.78 to 0.45/  -0.38 to 0.80 | |
| **Outcome Measures** | **Change from baseline to follow-up (18 weeks)**  **for NFE / NFS** | | | | | | | | |
|  | M | | 95% CI | | % -age | | Hedge’s g | | 95% CI |
| Hamilton Depression Rating Scale | -9.65/  -11.53 | | -6.25 to -13.06/  -8.61 to -14.45 | | -48/-59 | | -1.57 /  -2.06 | | -2.14 to -1.04/  -3.72 to -0.96 |
| Hospital Anxiety and Depression Scale (Anxiety Subscale) | -3.54/  -5.07 | | -0.82 to -6.25/  -3.19 to -6.94 | | -30/ -39 | | -0.72 /  -1.40 | | -1.19 to -0.03/  -1.84 to -1.03 |
| Hospital Anxiety and Depression Scale (Depression Subscale) | -4.38/  -4.33 | | -1.41 to -7.36/  -2.21 to -6.46 | | -35/-34 | | -0.82 /  -1.06 | | -1.35 to -0.37/  -1.43 to -0.71 |
| Behavioral Inhibition System | 1.62/  1.33 | | -0.82 to 4.05/  -0.43 to 3.1 | | 12/9 | | 0.37 /  0.38 | | -0.17 to 0.82/  -0.11 to 0.88 |
| Behavioral Activation System | -2.00/  2.67 | | -5.65 to 1.65/  -2.27 to 7.61 | | -6/7 | | -0.30 /  0.27 | | -1.19 to 0.33/  -0.25 to 0.67 |
| Quality of Life Scale | 13.31/  17.47 | | 7.47 to 19.15/  10.48 to 24.45 | | 24/32 | | 1.26 /  1.30 | | 0.68 to 2.00/  0.68 to 2.00 |
| Thought Control Questionnaire | 2.77/  -0.20 | | -3.70 to 9.24/  -5.38 to 4.98 | | 5/0 | | 0.24 /  -0.02 | | -0.39 to 0.70/  -0.56 to 0.50 |
| Thought Control Ability Questionnaire | 9.15/  10.73 | | 1.52 to 16.79/  4.1 to 17.37 | | 16/20 | | 0.66 /  0.84 | | 0.11 to 1.15/  0.34 to 1.35 |
| Self-Efficacy Scale, General Self-Efficacy Subscale | 4.15/  8.93 | | -1 to 9.31/  4.27 to 13.60 | | 9/18 | | 0.45 /  1.00 | | -0.12 to 1.06/  0.42 to 1.46 |
| Self-Efficacy Scale, Social Self-Efficacy Subscale | 0.69/  4.93 | | -1.74 to 3.12/  2.39 to 7.48 | | 4/36 | | -0.16 /  1.01 | | -0.46 to 0.63/  0.55 to 1.56 |
| EuroQol research foundation questionnaire | -1.73/  -1.85 | | -0.57 to -2.88/  -0.89 to -2.81 | | -15/-18 | | - 0.88 /  -1.06 | | -1.44 to -0.50/  1.83 to -0.49 |

**Table S3:** Additional details concerning whole-brain activity during neurofeedback blocks in the NFE and NFS group (positive x-coordinates denote right side of the brain).

| Anatomical label | Mean ± SD coordinates, X/Y/Z | Mean t-value | Mean p-value | Cluster size, mm^3^ |
| --- | --- | --- | --- | --- |
| **NFE Activation** |  |  |  |  |
| Cerebellum, Uvula | 17±15/-68±5/-23±3 | 4.666 | 0.0004 | 2251 |
| Caudate nucleus (body) | 15±3/25±2/11±3 | 5.189 | 0.0002 | 630 |
| Insula/ IFG | -29±14/20±6/7±9 | 5.209 | 0.0002 | 7584 |
| Thalamus (VAN) | 13±5/-5±5/-5±5 | 4.387 | 0.0006 | 1008 |
| Brainstem/midbrain | 2±7/-22±4/-11±2 | 4.588 | 0.0004 | 1834 |
| Posterior Cingulate/ Retrosplenial Cortex | -8±3/-54±9/7±5 | 4.707 | 0.0004 | 3765 |
| SMA | -5±3/6±6/51±8 | 4.684 | 0.0004 | 2137 |
| Thalamus (VLN) | -18±3/-9±7/11±4 | 4.825 | 0.0003 | 2194 |
| Hippocampus | -25±4/-12±7/-13±4 | 4.615 | 0.0004 | 2004 |
| Entorhinal cortex | -26±4/8±4/-10±3 | 4.570 | 0.0005 | 726 |
| Extrastriate cortex | -35±2/-57±3/12±5 | 4.516 | 0.0005 | 1177 |
| **NFE Deactivation** |  |  |  |  |
| IPL | 49±6/-52±10/39±5 | -4.422 | 0.0006 | 2165 |
| DLPFC | 44±3/31±5/26±5 | -4.483 | 0.0005 | 1365 |
| Superior frontal gyrus | 30±5/18±4/50± | -4.510 | 0.0005 | 1891 |
| **NFS Activation** |  |  |  |  |
| PPA | 29±4/-39±5/-14±5 | 4.592 | 0.0005 | 2518 |
| Posterior Cingulate | 15±4/-45±3/10±3 | 4.393 | 0.0006 | 783 |
| Lingual Gyrus | 13±3/-73±5/-10±4 | 4.456 | 0.0005 | 1613 |
| Posterior Cingulate | -3±5/-49±4/5±6 | 4.580 | 0.0005 | 2519 |
| PPA | -29±4/-35±5/-16±5 | 4.518 | 0.0005 | 3219 |
| Insula/ IFG | -37±3/22±3/-1±2 | 4.373 | 0.0006 | 493 |
| Premotor Cortex | -41±3/2±1/46±4 | 4.356 | 0.0006 | 529 |
| **NFS Deactivation** |  |  |  |  |
| Temporoparietal Junction | 54±2/-49±3/28±4 | -4.371 | 0.0006 | 672 |

**Table S4:** Additional details concerning whole-brain activity during neurofeedback blocks for between factor (group difference NFE>NFS) (positive x-coordinates denote right side of the brain).

| Anatomical label | Mean ± SD coordinates, X/Y/Z | Mean t-value | Mean p-value | Cluster size, mm^3^ |
| --- | --- | --- | --- | --- |
| **NFS > NFE** |  |  |  |  |
| VLPFC | 51±2/31±2/12±2 | -3.545 | 0.0006 | 362 |
| MTG | 43±5/-71±4/25±4 | -4.239 | 0.0002 | 2254 |
| Premotor Cortex | 27±9/0±6/49±4 | -3.871 | 0.0003 | 4180 |
| SPL | 21±8/-61±6/49±6 | -3.739 | 0.0004 | 4757 |
| Precentral Gyrus | 36±2/4±4/22±2 | -3.820 | 0.0003 | 629 |
| PPA, right | 26±3/-36±2/-14±3 | -3.957 | 0.0003 | 731 |
| Lingual Gyrus | 13±3/-73±5/-7±4 | -3.789 | 0.0004 | 1224 |
| Cingulate | 15±3/-52±4/10±5 | -3.683 | 0.0004 | 1191 |
| Precuneus | -16±7/-64±4/47±7 | -3.833 | 0.0004 | 3427 |
| MTG | -40±9/-75±6/24±7 | -3.832 | 0.0003 | 3069 |
| PPA, left | -26±3/-39±4/-13±4 | -3.762 | 0.0004 | 1340 |
| MFG (BA46) | -44±4/34±5/16±5 | -4.141 | 0.0002 | 3500 |
| MFG (BA9) | -52±3/14±4/30±4 | -3.975 | 0.0003 | 607 |
| IFG | -61±3/38±3/-12±2 | -3.803 | 0.0003 | 412 |
| **NFE>NFS** |  |  |  |  |
| MTG | 60±3/-14±2/-10±3 | 3.988 | 0.0003 | 703 |
| Insula | -29±3/-40±6/20±7 | 3.613 | 0.0005 | 1160 |
| ITG | -52±3/-5±2/-28±3 | 3.371 | 0.0004 | 412 |

**Anatomical label abbreviations in order of appearance.** IFG: Inferior Frontal Gyrus; VAN: Ventral Anterior Nucleus of the Thalamus; SMA: Supplementary Motor Area; IPL: Inferior Parietal Lobe; VLN: Ventral Lateral Nucleus of the Thalamus; DLPFC: Dorsolateral Prefrontal Cortex; SFG: Superior Frontal Gyrus; MTG: Middle Temporal Gyrus; SPL: Superior Parietal Lobe; MFG: Middle Frontal Gyrus; ITG: Inferior Temporal Gyrus. BA: Brodmann area.

**Figure S1.** Target voxel selection based on visual localizer task for NFE (top) and NFS (bottom) group in sagittal (left), transversal (center) and coronal (right) view.

**Supplementary References**

Delacre M, Lakens D, Leys C (2017). Why Psychologists Should by Default Use Welch’s t-test Instead of Student’s t-test. *Int Rev Soc Psychol* **30**: 92.

Eklund A, Nichols TE, Knutsson H (2016). Cluster failure : Why fMRI inferences for spatial extent have inflated false-positive rates. doi:10.1073/pnas.1602413113.

Kirsch I, Deacon BJ, Huedo-Medina TB, Scoboria A, Moore TJ, Johnson BT (2008). Initial Severity and Antidepressant Benefits: A Meta-Analysis of Data Submitted to the Food and Drug Administration. *PLoS Med* **5**: e45.

Lancaster JL, Woldorff MG, Parsons LM, Liotti M, Freitas CS, Rainey L, *et al* (2000). Automated Talairach Atlas labels for functional brain mapping. *Hum Brain Mapp* **10**: 120–131.

Peirce JW (2007). PsychoPy-Psychophysics software in Python. *J Neurosci Methods* **162**: 8–13.

Schuirmann D (1987). A comparison of the two-one side test procedures and the power approach for assessing the equivalence of average bioavailability. *J Pharmacokinet Biopharm* **15**: 657–681.
