# Supplementary figures and images for: Targeting the affective brain—a randomized controlled trial of real-time fMRI neurofeedback in patients with depression
Source: Neuropsychopharmacology. 2018 Jun 23;43(13):2578–85. doi: 10.1038/s41386-018-0126-5 (PMC6186421; doi:10.1038/s41386-018-0126-5)

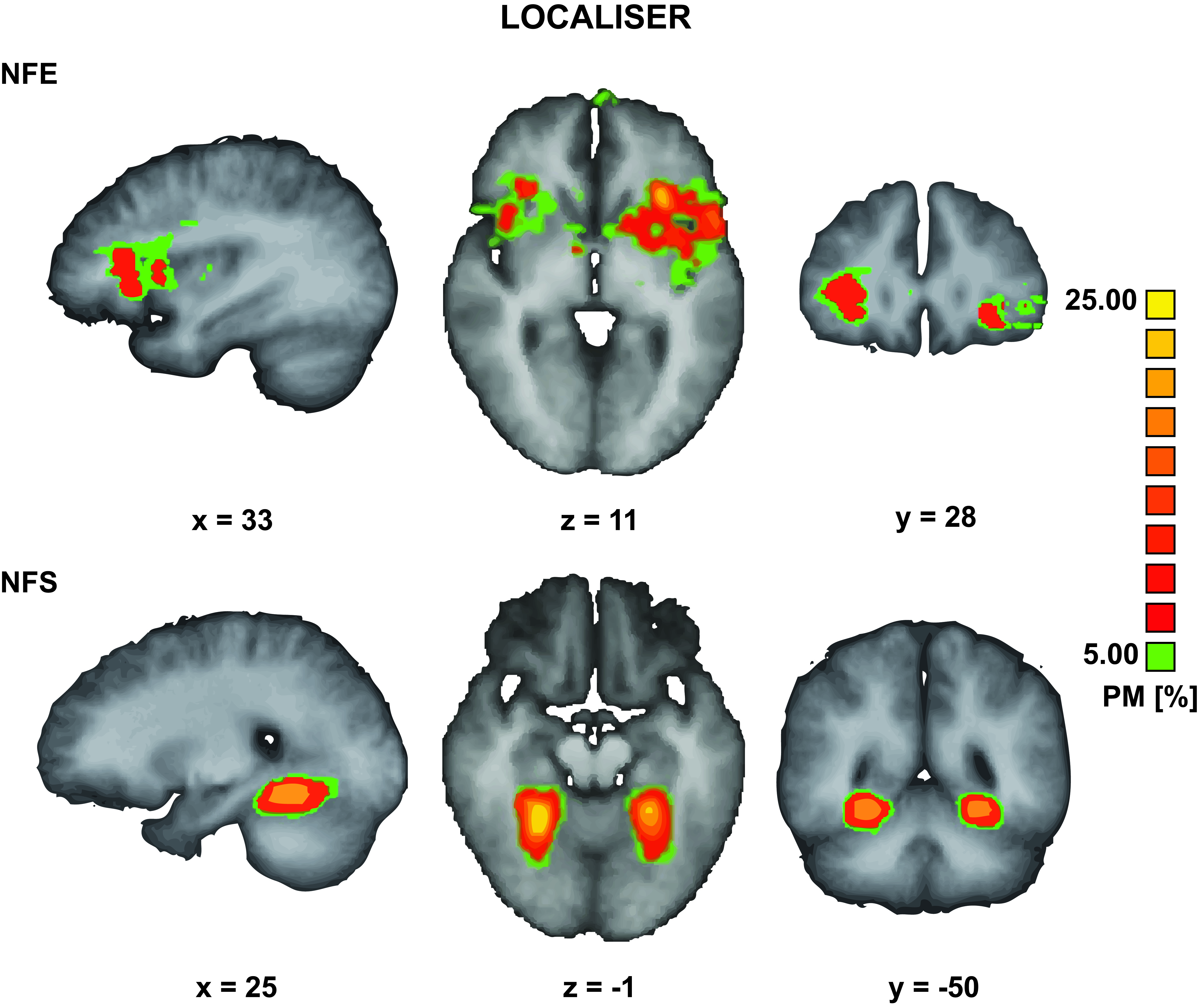

Supplement: Supplementary file 2 — Supplementary figure 1 [file 41386_2018_126_MOESM2_ESM.jpg]
